# Association of single nucleotide polymorphisms in the fatty acid synthase, LOC514211, and fat mass and obesity-associated genes with milk traits in Indonesian-Holstein dairy cattle

**DOI:** 10.14202/vetworld.2019.1160-1166

**Published:** 2019-07-30

**Authors:** Amalia Puji Rahayu, Tety Hartatik, Agung Purnomoadi, Edy Kurnianto

**Affiliations:** 1 Department of Animal Science, Faculty of Animal and Agricultural Sciences, Diponegoro University, Semarang 50275, Indonesia; 2 Agriculture, Fisheries and Food Center of Semarang Regency, Ungaran 50514, Indonesia; 3 Department of Animal Breeding and Reproduction, Faculty of Animal Sciences, Universitas Gadjah Mada, Yogyakarta 55281, Indonesia

**Keywords:** fat mass and obesity-associated, fatty acid synthase, Indonesian-Holstein cattle, LOC514211, milk traits

## Abstract

**Aim:**

The study aimed to identify fatty acid synthase (FASN), LOC514211, and fat mass and obesity-associated (FTO) gene polymorphisms and to investigate their associations with milk traits in an Indonesian-Holstein dairy cow population.

**Materials and Methods:**

A total of 100 Indonesian-Holstein cows consisting of 50 heads (0^th^ generation; G_0_) and 50 heads of their daughters (1^st^ generation; G_1_) were used. Polymerase chain reaction-restriction fragment length polymorphism was performed to genotype three single nucleotide polymorphisms: rs41919985 in the FASN gene, rs42688595 in the LOC514211 gene, and g.1371T>A in the FTO gene.

**Results:**

FASN rs41919985 was associated with milk protein percentage (p<0.05), FTO g.1371T>A was associated with milk fat percentage (p<0.05), and LOC514211 rs42688595 was not associated with any trait (p>0.05). Heterozygote variants showed a higher protein percentage for FASN and the highest fat percentage for FTO. These associations were consistent in the G_0_ and G_1_ populations.

**Conclusion:**

Our results indicate that the milk protein and fat percentages can be improved by increasing the frequency of the AG genotype of FASN and the AT genotype of FTO, respectively.

## Introduction

At present, dairy farmers are concerned with not only the milk yield but also the milk quality [1] because it affects the selling price to the milk processing industry. One method used to improve milk quality is to increase the genetic quality of cattle through selection [2]. For conventional selection, it is necessary to have the records of the parents and offspring. On the contrary, when phenotypic data are not available, genomic selection can be used to predict each individual’s genomic value, which can shorten generation intervals [3]. Schaeffer [4] stated that genomic selection can increase the efficiency of genetic gains per year by up to 50% and reduce 92% of the operational costs for progeny testing. Furthermore, a single nucleotide polymorphism (SNP) at the DNA level can be used for marker-assisted selection to select cattle with superior qualities [5].

Considering the benefits of genomic selection, it is very important to identify the SNPs in candidate genes responsible for milk traits. The fatty acid synthase (FASN), LOC514211, and fat mass and obesity-associated (FTO) genes are located on chromosomes 19, 13, and 18, respectively, in bovines. The FASN gene plays a role in fatty acid synthesis in the malonyl-coenzyme a pathway [6], while the FTO gene regulates the neurotrophin signaling pathway in the formation of milk fat [7]. The role of LOC514211 has not been definitively determined, but Anggraeni *et al*. [8] found that this gene was associated with milk fat yield. Therefore, these genes were chosen as candidate genes and were predicted to be associated with milk traits, especially milk fat yield. Research on the FASN gene has produced inconsistent results. FASN rs41919985 (which is the same as FASNg.17924A>G) was reported to affect milk fat content in the study by Schennink *et al*. [9], but no effect was found on in a study by Matsumoto *et al*. [10]. Meanwhile, there has been limited research on the other candidate genes, LOC514211 and FTO. Research on the FTO gene has mostly been conducted in humans [11,12] and meat livestock [13,14], with few studies in dairy cattle [7]. The SNP in the FTO gene analyzed in this study (which is the same as FTOg.12550T>A) was a novel SNP identified by Chung [14] in Hanwoo (Korean cattle). In Indonesian-Holstein cattle, some genetic markers have been studied [15-17], but to date, there have been no reports on the three candidate genes selected for the present study. Moreover, most studies on SNP markers only used individual records of dams without confirmation in the next generation [8,18]. Previous studies on various genetic markers have produced very different results in various populations. For example, in the study by Maylinda [15], a gene polymorphism in an imported Holstein cow population was significantly associated with milk traits, while the polymorphism in a local Holstein cow population was not, although the analyses were conducted in the same area. Therefore, different polymorphisms may be found in different generations, and their effects on milk traits can also be different.

Based on this background information, for an SNP to be considered a strong genetic marker, it is necessary to confirm its association in the parents (0^th^ generation; G_0_) and their daughters (1^st^ generation; G_1_). This study was designed to identify polymorphisms in the FASN, LOC514211, and FTO genes and to investigate their associations with milk traits in Indonesian-Holstein dams and their daughters.

## Materials and Methods

### Ethical approval

The protocol was carried out according to the standard rule of animal treating as appointed in the Republic of Indonesia’s law, number 41, 2014, as regard husbandry and animal health. This study was approved by Animal Ethics Committee in Faculty of Animal and Agricultural Sciences, Diponegoro University, Indonesia.

### Study area

This study was performed at the Baturraden Cattle Breeding and Forage Centre (BBPTUHPT Baturraden), Purwokerto, Central Java, Indonesia. DNA analyses were conducted at the Laboratory of Animal Breeding and Genetics, Universitas Gadjah Mada, Yogyakarta, Indonesia.

### Data collection

In this study, we focused on two generations on the same farm; thus, the availability of samples was limited. We used 100 samples (50 pairs of G_0_-G_1_; G_1_ was the daughter of G_0_) that were still available on the farm and met the criteria of having individual first lactation milk yield and milk composition records. In fact, other studies on gene association and polymorphism used smaller sample sizes [16,19-21]. DNA from the samples was isolated from cow tail vein blood using a DNA Extraction Kit (gSYNC^™^, Geneaid, Taiwan) following the manufacturer’s protocol. The studied parameters were milk fat percentage, total milk yield, milk fat yield (=fat%×milk yield), milk protein percentage, and milk protein yield (=protein%×milk yield).

### DNA amplification and genotyping

Based on the Holstein cattle DNA sequence in GenBank, three primer pairs were applied to amplify fragments of the candidate genes (FASN, LOC514211, and FTO) (Table-1) [8,18]. These candidate genes were selected as markers because they are involved in fat synthesis [6-8]. Because milk fat has a genetic correlation with milk yield and milk protein [22], these candidate genes were also used to assess these traits. The polymerase chain reaction (PCR) protocol was performed in a 25 μL volume, including 2 μL genomic DNA, forward and reverse primers (0.5 μL each), 12.5 μL MyTaq HS Red Mix (Bioline, UK), and 9.5 μL double-distilled water (ddH_2_O). The following program was used on a PCR machine (Advanced Primus 25, Peqlab, Germany): Predenaturation at 94°C for 5-10 min followed by 34-36 cycles consisting of denaturation at 94°C for 30 s; annealing at 60°C for 30 s for FASN, at 58.8°C for 45 s for LOC514211, or at 57°C for 20 s for FTO; extension at 72°C for 30-60 s; and a final extension at 72°C for 5-25 min. The products were analyzed using the restriction fragment length polymorphism (RFLP) method and first digested using restriction enzymes (Thermo Scientific, USA) in a 15 μL volume consisting of 4 μL PCR product; 1.5 μL buffer; 0.5 μL MscI for FASN, 0.3 μL TaqI for LOC514211, or 0.3 μL HpyCH4III for FTO; and ddH_2_O to the final volume. They were incubated at 37°C for 2 h for FASN or at 65°C for 3 h for LOC514211 and FTO [8,14,18]. The digested results were examined on a 3% agarose gel (stained with ethidium bromide) after 1 h of electrophoresis (50 V) in ×1 Tris-borate-EDTA buffer. The fragments were compared with a 50 bp DNA ladder (HyperLadder^™^, Bioline, UK). The representative results of each genotype were sequenced by PT. Genetika Science, Jakarta.

**Table 1:** Primer pairs used to amplify gene fragments.

Gene	Primer (5¢ → 3¢)	PCR product size (bp)	GenBank accession No.	References
FASN	F=AGAGCTGACGGACTCCACAC R=CTGCATGAAGAAGCACATGG	697	AF285607	[18]
LOC 514211	F=ACGGTGTTTGGGTTCCTG R=CTGTCTTGCCCTGTTTCG	352	rs42688595	[8]
FTO	F=TGCAAAGTACAATGAGGCCG R=CCCCATGCCAAAATACGGTT	301	HM777022	Designed by Primer 3 software

PCR=Polymerase chain reaction, FASN=Fatty acid synthase, FTO=Fat mass and obesity-associated, F=Forward, R=Reverse

### Statistical analysis

The allele and genotype frequencies were analyzed using the Chi-square test [17]. The general linear model procedure in the Statistical Package for the Social Sciences (SPSS) version 20 (IBM, USA) was performed to verify the association of the SNPs with traits as follows: Y_ij_=μ+τ_i_+ε_ij_, where Y_ij_=The analyzed trait, μ=General mean, τ_i_=i^th^ genotype effect, and ε_ij_=Random error effect. The G_0_ and G_1_ trait performances were compared using t-test. Environment factors were not included in the model because (1) G_0_ and G_1_ were similarly managed on the same farm, (2) the milk yield was corrected for the calving age using a correction factor [23], and (3) there were no extreme seasonal changes. Note that, the similar environment had little effect on the production traits (correlation value 0.14), and Vanvleck and Barr [24] and Mulder *et al*. [25] stated that when studying quantitative genetics, it could be assumed that the environmental variances among contemporary groups (e.g., calving year-season) could be ignored.

## Results

### Allele frequencies of the SNPs

**Figure-1:**
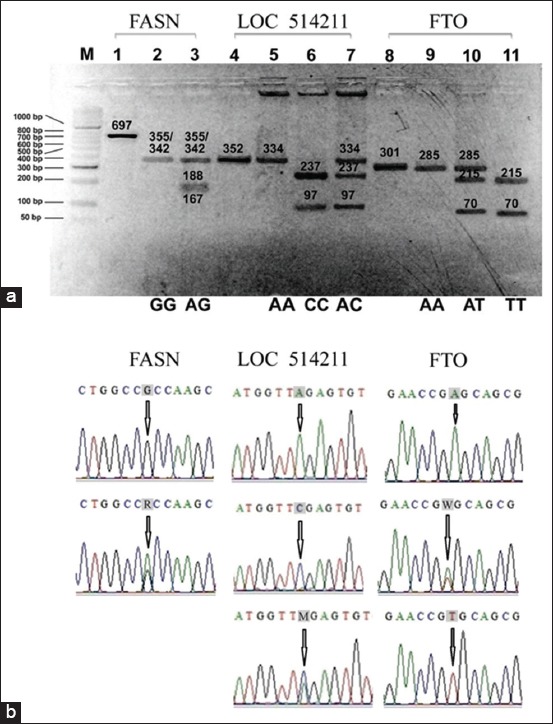
Representative results of polymerase chain reaction-restriction fragment length polymorphism (PCR-RFLP) following (a) electrophoresis. All numbers in the box in units of bp. M: 50 bp DNA marker; 1, 4, and 8: PCR products; 2-3: PCR-RFLP FASN-MscI; 5-7: PCR-RFLP LOC514211-TaqI; 9-11: PCR-RFLP FTO-HPyCH4III; (b) sequencing. Shaded letters indicate single nucleotide polymorphism positions in chromatograms. R=G or A, M=A or C, W=A or T.

The polymorphisms detected in PCR-RFLP fragments of the Indonesian-Holstein FASN, LOC514211, and FTO genes are shown in Figure-1. There were only two FASN rs41919985 genotypes found in this study, GG (342 bp/355 bp) and AG (167 bp/188 bp/342 bp/355 bp), while three genotypes each were found for LOC514211 rs42688595, AA (334 bp), CC (97 bp/237 bp), and AC (97 bp/237 bp/334 bp), and FTO g.1371T>A, AA (285 bp), TT (70 bp/215 bp), and AT (70 bp/215 bp/285 bp).

The genotype and allele frequencies of the SNPs are presented in Table-2. The G allele frequency of FASN rs41919985 was higher than the A allele frequency. G_0_ contained 79% G allele and 21% A allele, whereas G_1_ contained 84% G allele and 26% A allele. The frequency of the C allele (0.51) of LOC514211 rs42688595 was higher than the frequency of the A allele (0.49). CC was the most frequent genotype (0.42), followed by AA (0.39) and AC (0.19). The most common genotype of FTO g.1371T>A was the AA genotype.

**Table 2:** Genotype and allele frequencies of SNPs.

SNP	Generation	n	Genotypes			Alleles		χ^2^ test
	
			AA	AG	GG	A	G	
FASN	G_0_	50	0.00	0.42	0.58	0.21	0.79	E (χ^2^=3.53)
rs41919985	G_1_	50	0.00	0.32	0.68	0.16	0.84	E (χ^2^=1.81)
	All samples	100	0.00	0.37	0.63	0.19	0.81	
			AA	AC	CC	A	C	
LOC514211	G_0_	50	0.38	0.20	0.42	0.48	0.52	D (χ^2^=17.96)
rs42688595	G_1_	50	0.40	0.18	0.42	0.49	0.51	D (χ^2^=20.47)
	All samples	100	0.39	0.19	0.42	0.49	0.51	
			AA	AT	TT	A	T	
FTO	G_0_	50	0.72	0.22	0.06	0.83	0.17	E (χ^2^=2.43)
g. 1367A>T	G_1_	50	0.86	0.08	0.06	0.90	0.10	D (χ^2^=15.43)
	All samples	100	0.79	0.15	0.06	0.87	0.13	

FASN=Fatty acid synthase, FTO=Fat mass and obesity-associated, n=The number of samples, G_0_=0^th^ generation, G_1_=1^st^ generation, All samples=Both G_0_ and G_1_, D=Disequilibrium (χ^2^>χ^2^ table), E=Equilibrium (χ^2^<χ^2^ table), χ^2^ table_(0,05; df=1)_=3,841, SNP=Single nucleotide polymorphism

### Association of the SNPs with milk traits

As shown in Table-3, FASN rs41919985 was associated with the milk protein percentage (p=0.002-0.048). Milk produced from the GG genotype was characterized by a lower protein percentage (2.90%) than milk produced from the AG genotype (3.08%). As shown in Table-4, LOC514211 rs4268859 did not affect the studied traits. As presented in Table-5, FTO g.1371T>A was associated with fat percentage. Cows with the AT genotype had a significantly higher fat percentage (4.41-4.74%) than cows with the TT genotype (3.17-3.92%). As shown in Table-6, the milk fat and protein percentages of G_1_ (3.81% and 2.87%, respectively) were lower than those of G_0_ (4.50% and 3.00%, respectively).

**Table 3:** Association of FASN rs41919985 with milk traits.

Parameter	Generation	Genotype				p-value

		GG		AG		
	
		n	Y¯±SEM	n	Y¯±SEM	
Milk yield (x1000 kg)	G_0_	29	4.17±0.23	21	3.63±0.29	0.146
	G_1_	34	4.01±0.23	16	4.25±0.38	0.579
	All samples	63	4.09±0.16	37	3.90±0.24	0.496
Fat (%)	G_0_	29	4.46±0.13	21	4.57±0.08	0.519
	G_1_	34	3.63±0.12	16	3.89±0.11	0.167
	All samples	63	4.05±0.09	37	4.22±0.10	0.451
Fat yield (kg)	G_0_	29	184.56±11.03	21	163.66±12.19	0.214
	G_1_	34	155.00±9.43	16	152.20±13.15	0.866
	All samples	63	168.61±7.37	37	158.71±8.88	0.403
Protein (%)	G_0_	29	2.96±0.02^b^	21	3.21±0.02^a^	0.002
	G_1_	34	2.85±0.05^b^	16	2.95±0.06^a^	0.048
	All samples	63	2.90±0.03^b^	37	3.08±0.03^a^	0.038
Protein yield (kg)	G_0_	29	123.34±6.81	21	110.49±9.00	0.251
	G_1_	34	114.09±6.55	16	122.17±10.68	0.505
	All samples	63	118.35±4.72	37	115.54±6.86	0.729

FASN=Fatty acid synthase, n=The number of samples, Y¯=Average value, SEM=Standard error of the mean, G_0_=0^th^ generation, G_1_=1^st^ generation, All samples=Both G_0_ and G_1_. ^ab^ Values with different superscripts letters within the same rows differ significantly (p<0.05)

**Table 4:** Association of LOC514211 rs42688595 with milk traits.

Parameter	Generation	Genotype						p-value

		AA		AC		CC		
		
		n	Y¯±SEM	n	Y¯±SEM	n	Y¯±SEM	
Milk yield (x1000 kg)	G_0_	19	4.28±0.27	10	3.58±0.38	21	3.82±0.31	0.337
	G_1_	20	4.17±0.34	9	4.62±0.46	21	3.79±0.27	0.287
	All samples	39	4.22±0.22	19	4.07±0.31	42	3.39±0.20	0.373
Fat (%)	G_0_	19	4.43±0.16	10	4.52±0.15	21	4.56±0.11	0.787
	G_1_	20	3.63±0.12	9	3.92±0.14	21	3.92±0.16	0.268
	All samples	39	4.02±0.12	19	4.24±0.12	42	4.24±0.11	0.304
Fat yield (kg)	G_0_	19	188.67±13.40	10	161.87±17.51	21	170.75±13.08	0.446
	G_1_	20	152.15±13.95	9	176.70±14.89	21	146.28±10.41	0.364
	All samples	39	169.94±10.00	19	168.90±11.42	42	158.51±8.47	0.632
Protein (%)	G_0_	19	3.00±0.03	10	3.00±0.06	21	2.99±0.02	0.844
	G_1_	20	2.83±0.06	9	2.91±0.10	21	2.89±0.06	0.695
	All samples	39	2.92±0.03	19	2.96±0.06	42	2.94±0.03	0.773
Protein yield (kg)	G_0_	19	128.81±8.38	10	132.72±11.30	21	113.24±9.03	0.281
	G_1_	20	117.85±10.09	9	133.03±12.43	21	108.56±7.34	0.168
	All samples	39	123.19±6.56	19	119.42±8.68	42	110.90±5.76	0.356

n=The number of samples, Y¯=Average value, SEM=Standard error of the mean, G_0_=0^th^ generation, G_1_=1^st^ generation, All samples=Both G_0_ and G_1_. ^ab^ Values with different superscripts letters within the same rows differ significantly (p<0.05)

**Table 5:** Association of FTO g. 1367A>T with milk traits.

Parameter	Generation	Genotype						p-value

		AA		AT		TT		
		
		n	Y¯±SEM	n	Y¯±SEM	n	Y¯±SEM	
Milk yield (x1000 kg)	G_0_	36	4.06±0.21	11	3.63±0.38	3	4.37±0.07	0.528
	G_1_	43	4.06±0.21	4	4.46±0.95	3	4.03±0.64	0.862
	All samples	79	4.06±0.15	15	3.85±0.37	6	4.20±0.30	0.818
Fat (%)	G_0_	36	4.48±0.10^ab^	11	4.74±0.09^a^	3	3.92±0.40^b^	0.049
	G_1_	43	3.84±0.09^ab^	4	4.41±0.19^a^	3	3.17±0.28^b^	0.029
	All samples	79	4.13±0.08^ab^	15	4.52±0.15^a^	6	3.54±0.28^b^	0.022
Fat yield (kg)	G_0_	36	179.04±9.86	11	166.49±19.33	3	170.72±15.10	0.819
	G_1_	43	154.95±8.36	4	166.39±29.50	3	125.60±16.65	0.597
	All samples	79	165.93±6.50	15	166.46±15.67	6	148.16±14.24	0.760
Protein (%)	G_0_	36	2.99±0.02	11	3.02±0.02	3	2.94±0.08	0.527
	G_1_	43	2.89±0.04	4	2.80±0.10	3	2.67±0.11	0.270
	All samples	79	2.94±0.03	15	2.96±0.04	6	2.81±0.09	0.276
Protein yield (kg)	G_0_	36	120.50±6.53	11	106.79±12.79	3	128.08±2.13	0.539
	G_1_	43	116.73±6.07	4	123.78±25.95	3	106.51±13.59	0.854
	All samples	79	118.45±4.42	15	111.32±11.32	6	117.30±7.82	0.813

FTO=Fat mass and obesity-associated, Y¯=Average value, SEM=Standard error of the mean, G_0_=0^th^ generation, G_1_=1^st^ generation, All samples=Both G_0_ and G_1_. ^ab^Values with different superscripts letters within the same rows differ significantly (p<0.05)

**Table 6:** Comparison of milk traits between G_0_ and G_1_.

Parameter	Generation (Y¯±SEM)		p-value

	G_0_ (n=50)	G_1_ (n=50)	
Milk yield (x1000 kg)	3.94±0.18	4.09±0.20	0.588
Fat (%)	4.50±0.08^a^	3.81±0.09^b^	<0.001
Fat yield (kg)	175.78±8.24	154.11±7.60	0.054
Protein (%)	3.00±0.02^a^	2.87±0.04^b^	0.002
Protein yield (kg)	117.94±5.48	116.68±5.58	0.872

Y=Average value, SEM=Standard error of the mean, n=The number of samples, G_0_=0^th^ generation, G_1_=1^st^ generation.

ab Values with different superscripts letters within the same rows differ significantly (p<0.05)

## Discussion

### Allele frequencies of the SNPs

The genotypes were determined in this study by observing the DNA bands. Different fragments were obtained because if a nucleotide base undergoes a mutation, the cutting location for a restriction enzyme will change, so an enzyme might cut normal DNA fragments and mutated DNA fragments in different places [15]. Thus, after the mutation, the fragments formed will be different from the fragments produced with normal DNA.

FASN rs41919985 in the population studied was polymorphic with two genotypes, namely, AG and GG. Different from the results of this study, in which the AA genotype was not found, a previous study found three genotypes (GG, AG, and AA), but the AA genotype only appears at a low frequency of 0.06 in Turkey Anatolian Red cattle [26] and 0.08 in Chinese Holstein cattle and Fleckvieh bulls [18,27]. The AA genotype was reported at a higher frequency (0.28) in Dutch Holstein cattle [9]. Thus, the absence of AA genotypes in this study was not because the AA genotype is lethal but probably because the G allele is a common allele in various populations. Six samples of frozen bull semen that had been used (the sires of G_1_) were tested, and all were the GG genotype. According to An *et al*. [28], genotypes may not occur in a certain population because (1) individual performance was adversely affected by the missing genotype, and the animals with missing genotypes were excluded from the breeding process and (2) the missing genotype is rarer. Because the AA genotype was not found in G_0_ or the sire population, there was no AA genotype in G_1_, and the frequency of the A allele decreased. Therefore, the frequency of the GG genotype became higher in G_1_.

The order of the LOC514211 rs4268859 genotype frequencies from the highest to the lowest (CC, AA, and AC) in this study was different from that reported by Anggraeni *et al*. [8] in Chinese Holstein cattle: AA (0.46), AC (0.33), and CC (0.21). The FTO g.1371T>A AA genotype frequency of 0.72-0.86 found in this study was much higher than that detected in Korean Hanwoo cattle (0.47) [13], but the lowest genotypic frequency (TT) was similar in both studies (0.13 vs. 0.08).

The results of the studies mentioned above indicate that the allele frequencies observed in most studies of polymorphisms were specific for different cattle breeds or different cattle populations. Based on the expected versus observed genotype frequencies, LOC 514211 rs42688595 and FTO g.1371T>A were not in Hardy–Weinberg genetic equilibrium probably because of the limitation of the population size, the selection program for breeding, the mixing of the population with imported cows, and the migration of cows in BBPTUHPT Baturraden. According to Andrews [29], selection, non-random mating, and migration can cause changes in the frequency of certain genes, so one or more standard assumption for Hardy–Weinberg equilibrium might be broken.

### Association of the SNPs with milk traits

The results of this study showed that the AG genotype of FASN rs41919985 resulted in a higher protein percentage than the GG genotype. No association of the SNP FASN rs41919985 with milk traits was found in Japanese Holstein cattle [10]. Different results, however, were reported by Bartoň *et al*. [27] and Schennink *et al*. [9], as they found that this SNP affected the level of milk yield, fatty acid composition, and milk fat percentage. The association of this SNP with milk protein percentage was not discussed in these studies; nevertheless, an association with milk protein yield has been reported [30].

The ruminant udder cannot immediately use acetyl-coenzyme A derived from glucose in mitochondria. The tricarboxylic acid cycle is only used to produce nicotinamide adenine dinucleotide phosphate hydrogen (NADPH). In the presence of NADPH, malonyl-coenzyme A and acetyl-coenzyme A are converted to palmitic acid. This process is catalyzed by FASN [27]. SNP rs41919985 is found on exon 39 of the FASN gene [26] and is a missense mutation that results in an amino acid change from threonine (ACC) to alanine (GCC). This mutation likely affects the structure of the substrate-binding site and therefore, may result in differences in enzymatic regulation and different milk compositions [26,27]. The FASN gene likely affects protein percentage through a pleiotropic action. Protein percentage is genetically positively correlated to the fat percentage with a moderate to high correlation coefficient (0.16-0.55) [22]. The milk protein percentage can be improved by increasing the frequency of the AG genotype. Considering that a mating design where AG is crossed with AG will also produce the GG and AA genotypes, we recommend that the offspring with the GG genotype be culled from the population and that the AA genotype should be further studied to determine its association with milk traits.

The LOC514211 gene is an uncharacterized protein-coding gene [8]. The finding of no significant association for LOC 514211 rs42688595 in this study was not in line with those of previous genome-wide association studies based on 60k SNP-Chips [31] and PCR-RFLP in other populations [8], which found that this SNP affected milk and fat yield. The role of LOC514211 is not clear, and no previous study has explored the effect of the LOC514211 gene on milk traits.

At present, there are no available data on FTO g.1371T>A in dairy cattle in literature. However, this SNP has been studied in beef cattle (Hanwoo) [13], and the findings indicated that the AA genotype was correlated to a higher marbling score than the TT genotype. Similarly, in this study, the AT and AA genotypes correlated to a higher milk fat percentage than the TT genotype. In both studies, the TT genotype was rarely found, so it is not recommended for selection. If the TT genotype is ignored because of its low incidence and statistical testing are performed between only the AA and AT genotypes, cows with the AT genotype have significantly higher fat content than those with the AA genotype (p=0.005), so we recommend the AT genotype for selection programs.

The FTO gene encodes Fe(II)-and 2-oxoglutarate-dependent dioxygenases. The FTO gene is the most abundant in the hypothalamus, which is the energy balance control center [14]. Polymorphic variations in FTO may affect fatty acid metabolism, demethylation catalysis of nucleic acids, energy homeostasis and energy partitioning regulation, and fat tissue development [32]. The mutations in LOC514211 rs42688595 (CGA→AGA) and FTO g.1371T>A (CGU→CGA) are silent mutations that did not cause an amino acid change (in both cases, arginine is still produced). Although these are synonymous SNPs, they can influence gene function because synonymous SNPs may alter the structure, expression level, and function of proteins and affect mRNA splicing and stability [33].

Overall, the inconsistent results between our work and those of previous studies could be related to the background genes in the different breeds of cattle. G_1_ showed a slightly higher milk yield (4089 kg) than G_0_ (3943 kg), although this difference was not statistically significant. This increase in milk yield was the result of a selection program conducted at BBPTUHPT Baturraden, in which a certain proportion of the low-producing cows was culled. The lower fat and protein percentages of G_1_ may be due to negative genetic correlations between milk yield and fat percentage and between milk yield and protein percentage (−0.3-−0.5, respectively) [34]. However, although G_1_ have significantly lower fat and protein percentages than G_0,_ the associations between the SNPs of the three candidate genes and observed traits were consistent in the G_0_ and G_1_ populations, reinforcing the evidence indicating that the effects of the genes on G_0_ are in line with their effects on G_1_.

## Conclusion

The AG genotype of FASN rs41919985 and the AT genotype of FTOg.1371T>A were associated with higher milk protein and fat percentages, respectively, whereas LOC514211 rs42688595 was not associated with any of the examined traits. These associations were consistent in the G_0_ and G_1_ populations. It can be concluded that the SNPs FASN rs41919985 and FTOg.1371T>A can potentially be used as markers for the characteristics of milk protein and milk fat percentages, respectively.

## Authors’ Contributions

APR designed the experiment; performed data collection, DNA analysis, statistical analysis, and results in interpretation; and drafted the manuscript. TH, AP, and EK designed the experiment, supervised the fieldwork, interpreted the obtained results, and completed the critical revision of the manuscript. All authors read and approved the final manuscript.
